# How to support dental students in reading radiographs: effects of a gaze-based compare-and-contrast intervention

**DOI:** 10.1007/s10459-020-09975-w

**Published:** 2020-06-02

**Authors:** Thérése F. Eder, Juliane Richter, Katharina Scheiter, Constanze Keutel, Nora Castner, Enkelejda Kasneci, Fabian Huettig

**Affiliations:** 1 Leibniz-Institut für Wissensmedien, Schleichstraße 6, 72076 Tübingen, Germany; 2 University of Tübingen, Tübingen, Germany; 3 Department of Oral- and Maxillofacial Radiology, Centre for Dentistry, Oral Medicine, and Maxillofacial Surgery, University Hospital Tübingen, University of Tübingen, Tübingen, Germany; 4 Perception Engineering, Department of Computer Science, University of Tübingen, Tübingen, Germany; 5 Department of Prosthodontics, Centre for Dentistry, Oral Medicine, and Maxillofacial Surgery, University Hospital Tübingen, University of Tübingen, Tübingen, Germany

**Keywords:** Dental medicine, Eye movement visualization, Gaze-based intervention, Gaze behavior, Medical image interpretation, Radiology, Visual expertise

## Abstract

In dental medicine, interpreting radiographs (i.e., orthopantomograms, OPTs) is an error-prone process, even in experts. Effective intervention methods are therefore needed to support students in improving their image reading skills for OPTs. To this end, we developed a compare-and-contrast intervention, which aimed at supporting students in achieving full coverage when visually inspecting OPTs and, consequently, obtaining a better diagnostic performance. The comparison entailed a static eye movement visualization (heat map) on an OPT showing full gaze coverage from a peer-model (other student) and another heat map showing a student’s own gaze behavior. The intervention group (*N* = 38) compared five such heat map combinations, whereas the control group (*N *= 23) diagnosed five OPTs. Prior to the experimental variation (pre-test) and after it (post-test), students in both conditions searched for anomalies in OPTs while their gaze was recorded. Results showed that students in the intervention group covered more areas of the OPTs and looked less often and for a shorter amount of time at anomalies after the intervention. Furthermore, they fixated on low-prevalence anomalies earlier and high-prevalence anomalies later during the inspection. However, the students in the intervention group did not show any meaningful improvement in detection rate and made more false positive errors compared to the control group. Thus, the intervention guided visual attention but did not improve diagnostic performance substantially. Exploratory analyses indicated that further interventions should teach knowledge about anomalies rather than focusing on full coverage of radiographs.

## Introduction

Reading radiographs such as orthopantomograms, (OPTs, panoramic radiographs of the upper and lower mandible including dentition), is a standard diagnostic procedure in the daily work of dentists, but is an error-prone process (Stheeman et al. 1996). Undetected or misinterpreted anomalies can have serious consequences for patients. For instance, carotid calcifications in the soft tissues of the neck can potentially lead to a stroke (Friedlander et al. 2005; Tamura et al. 2005). To avoid these diagnostic errors, it is important to start training early. However, training targeted at improving students’ diagnostic performance is lacking (Kok et al. 2017). We therefore designed this study to evaluate a gaze-based training intervention for dental students.

Diagnostic errors in medical image interpretation can be classified into two groups (Gegenfurtner et al. 2017). Diagnosing a feature as abnormal although it does not represent an anomaly corresponds to a false positive error, whereas diagnosing a feature as normal although it represents an anomaly corresponds to a false negative error. False negative errors are particularly problematic, as health-threating situations are overlooked while false positive errors can be corrected in the further course of an albeit unnecessary treatment. False negative errors can be further classified into detection, recognition and decision-making errors (Al-Moteri et al. 2017; Donovan and Litchfield 2013; Kundel et al. 1978). Detection errors occur when an observer does not visually attend to, or overlook, an anomaly; these errors result from misguided perception processes (bottom-up process). Recognition errors occur when an observer attends to anomalies, but lacks knowledge about characteristic features of anomalies and healthy structures, thereby not recognizing the anomalies (top-down process). Decision-making errors occur when the observer fixates on the anomaly and recognizes ambiguous features, but decides against their clinical relevance (top-down process).

The frequency of these errors, which has been mostly investigated in chest radiographs using eye tracking (Donovan and Litchfield 2013; Kundel et al. 1978; Manning et al. 2004) and using radiographs and computer tomography (CT) images (Donald and Barnard 2012), differs. Donald and Barnard (2012) found 80% of errors were detection errors and the aforementioned eye-tracking studies showed a maximum of 35% detection errors. A possible explanation for this difference could be the types of images (radiographs and CTs), which are related to different anatomical areas. This explanation corresponds to findings of a meta-analysis by Gegenfurtner et al. (2011) suggesting that domain specificity and task characteristics influence visual search. Consequently, findings from studies conducted with a certain type of image and task cannot be directly transferred to other images and tasks.

To the best of our knowledge, only two other studies have investigated visual search in OPTs (Grünheid et al. 2013; Turgeon and Lam 2016) and none of them investigated error types. There are good reasons to assume that the frequency of error types in OPTs differs from that in chest radiographs. Chest radiographs typically indicate no more than five anomalies, which is rather a small number compared to anomalies that can be found in OPTs (Donovan and Litchfield 2013; Kundel et al. 1978). In the OPTs used in the present study, which were obtained from patients reporting no obvious complaints, there were up to 26 anomalies within one OPT. Consequently, the likelihood for detection errors is higher in OPTs due to the larger number of anomalies. In contrast with experts, dental students are likely to commit even more detection errors, because they need to apply a search-to-find method (Kundel et al. 2007; Nodine and Mello-Thoms 2000). Additionally, detection errors are more likely to occur for low-prevalence anomalies rather than high-prevalence anomalies. Low-prevalence anomalies are located more often in the periphery compared to the central areas of the oral cavity (Constantine et al. 2018; Vallo et al. 2010).

The different error types as well as visual search processes in image reading can be investigated by means of eye tracking (Kok and Jarodzka 2017a), where the gaze of a person inspecting a stimulus is recorded with a camera. The gaze is later analyzed with respect to its spatial and temporal characteristics, thereby allowing statements about which elements of the stimulus were looked at, when, and for how long. In these analyses, the gaze is further divided into separate events, namely, fixations and saccades. During a fixation, the gaze remains focused on one area of an image and information about this area can be processed (Just and Carpenter 1980; Kok and Jarodzka 2017a). Saccades are fast movements to re-position the eye and hence change the focus of attention. For these two types of events, various different eye tracking measures can be determined that provide important insights into a person’s gaze behavior. Commonly used eye tracking measures for visual search in medical images are the time to first fixation regarding a specific area of interest (AOI; e.g., an anomaly), total fixation time, the number of fixations, the number and length of saccades as well as image coverage (van der Gijp et al. 2017). The time to first fixation denotes the time it takes a person to first attend to an anomaly. Number of fixations and fixation time (duration of fixations) on AOIs typically reflect more intense processing of this area. The coverage denotes the degree to which a person has inspected an image by having fixated in multiple areas. In the present study, these measures were used to investigate the effectiveness of the intervention, thereby assuming that the intervention would improve diagnostic performance via changing students’ visual search behavior.

So far, there is little research describing and evaluating training approaches for improving visual interpretation of radiographs (Kok et al. 2017). Nevertheless, some systematic approaches for interpreting radiographs do exist. A systematic approach for OPTs is based on the division of the radiograph into four different regions of interest (Pasler 1991), which should all be inspected to prevent students from missing anomalies. Especially novices, who typically process only small parts of images (Jaarsma et al. 2014), should use a full coverage approach in order to detect all anomalies. Previous research has, however, shown that a full coverage training may not necessarily lead to better diagnostic performance. In a study by Kok et al. (2016) medical students were asked to mentally divide a radiograph into segments and then separately search in every segment, which did not yield better diagnostic performance. An explanation could be that the training was rather artificial, and possibly interrupted the students in their own strategies of searching for anomalies.

An innovative instructional method to enhance full image coverage is to illustrate adequate visual search behavior by showing how a role model (e.g., an expert or advanced learner) would perform these search processes. Here, eye tracking is not only used for measuring attentional processes, but also as an instructional tool (cf. Scheiter and Eitel 2016). Gaze-based modeling has been used effectively in various contexts to support learning (e.g., multimedia learning: Mason et al. 2015; clinical reasoning: Jarodzka et al. 2012). When applying eye movement modeling to diagnostic search tasks, the gaze behavior of a person (i.e., the model) searching for anomalies is visualized and displayed as training material to learners. The learners observe the model’s gaze behavior and are supposed to incorporate his/her behavior into their own repertoire of cognitive strategies (van Merriënboer and Kirschner 2007). Eye movement modeling has been shown to foster diagnostic performance (for chest radiographs: Litchfield et al. 2010; for PET/CT: Gegenfurtner et al. 2017). Against this backdrop, we used a model’s gaze to visualize full gaze coverage of a radiograph, which is expected to improve coverage—and in turn diagnostic performance—in students. We used static gaze visualizations (i.e., heat maps) where the model’s distribution of visual attention was visualized and superimposed onto the OPT. Thus, areas attended by the model were highlighted while the underlying structure and the rest of the image remained visible (cf. Jarodzka et al. 2012).

More important, not every model is equally helpful. The model-observer similarity effect states that learners are more likely to adopt the model’s behavior if s/he is perceived as being similar (Schunk 1987; Schunk and Hanson 1985). Accordingly, Krebs et al. (2019) found that students with low prior knowledge profited from eye movement modeling only if the models were introduced as peer-models but not as expert-models. Moreover, radiologist experts use search strategies that cannot be deployed by novices yet (i.e., global-focal search; Kundel et al. 2007; Nodine and Mello-Thoms 2000), who lack the necessary knowledge. In particular, experts require a quick glance at a suspicious area of an image only, whereas good performance in students is likely to be characterized by intense processing of all areas of an image. Thus, it is questionable whether students could learn from gaze visualizations obtained from an expert model (cf. van der Gijp et al. 2017). Therefore, we chose heat maps from other, more advanced students who showed full coverage of the OPTs and intense processing of all its areas as peer-models to guarantee a high model-observer similarity.

An approach that combines modeling with individualized learning is the compare-and-contrast approach (van Merriënboer and Kirschner 2007). Kok et al. (2013) showed that students who compared and contrasted chest radiographs indicating diseases against radiographs without diseases improved their diagnostic skills compared to students who only studied radiographs indicating diseases. Against this backdrop, in the present study we asked students to compare and contrast the gaze coverage of a peer-model with a gaze display of their own that had been recorded in an earlier trial to encourage more active processing of the model’s gaze display and to enhance the students’ understanding of systematic search.

### The present study

The goal of the study was to improve dental students’ diagnostic performance of reading OPTs by encouraging them to fully cover the image during visual inspection by means of a training. A full coverage of an OPT should help to avoid overlooking peripheral anomalies and thus reduce the number of detection errors. To support a full coverage, we combined two different instructional approaches within a gaze-based intervention. First, we presented students with a static gaze visualization obtained from a peer learner adjunct to their own gaze visualization. The peer-model’s gaze visualization served as reference standard to which the participants could compare their own search behavior. Second, we asked them to compare and contrast the two visualizations. The visualizations were heat maps, where more saturated colors indicated more attention to an area. The intervention group was contrasted with data from a business-as-usual control group, who took part only in the routine training offered to the dental students.

First, we hypothesized that the compare-and-contrast modeling intervention leads to a more complete visual search, which should be reflected in a more comprehensive coverage when inspecting radiographs. Thus, the coverage should increase in the intervention group from pre- to post-test, whereas the coverage in the control group should not change over time (Hypothesis 1).

Second, we expected the change in gaze behavior due to training to differ between anomalies located in peripheral areas and those in central areas (Hypotheses 2a–c). Consequently, we assumed a three-way interaction between time (pre- vs. post-test), intervention (intervention vs. control group), and location (peripheral vs. central). The number of fixations (Hypothesis 2a) and the fixation time (Hypothesis 2b) for peripheral anomalies should increase from the pre- to the post-test in the intervention group, but not in the control group. Additionally, we assumed that students in the intervention group, but not in the control group, would fixate on anomalies in the peripheral area in the post-test sooner than in the pre-test (Hypothesis 2c). No changes were expected for central anomalies for any of the gaze measures.

Because students in the intervention group were expected to show improved visual coverage of the OPTs, it was also assumed that they would conduct fewer detection errors, resulting in better diagnostic performance. Thus, the training should improve diagnostic performance from pre- to post-test as a function of anomaly location (three-way interaction: time × location × intervention). The diagnostic performance in the intervention group, but not in the control group, should increase from the pre- to the post-test especially for peripheral anomalies; fewer, if any, improvements were expected for central anomalies (Hypothesis 3).

## Methods

### Participants and design

78 dental students, who were either in their 7th or 9th semester, participated voluntarily in the experiment. At the Dental Medical School of the University of Tübingen, all dental students are requested to take part in a radiology course, where they are taught about radiation, imaging techniques, and radiograph interpretation in the 6th semester; this course includes massed practice of interpreting 100 images, mostly OPTs (cf. Richter et al., 2019). They graduate after the 10th semester. On average there are 22 students in each study cohort, with a new cohort starting each summer and winter term. Accordingly, when inviting 7th and 9th semester students in two consecutive terms to participate in the study, a full-scale survey would have contained 88 students—which was nearly achieved. Accordingly, our sample was reasonably representative of the overall population of dental medical students at these two study levels. As incentives, students received a 15€ book voucher and individual feedback regarding their performance and gaze behavior at the end of the semester. 14 students did not complete the whole experiment (i.e., they did not participate in either the pre-test or in the post-test session) and thus had to be excluded from data analyses. Data from 3 students were excluded due to technical problems. The control group consisted of 23 students in the 9th semester (*N* = 23 (16 female); mean age = 25.39 years, *SD* = 2.48). The intervention group consisted of 7th (*N* = 23 (11 female); mean age = 24.27 years, *SD* = 2.61) and 9th (*N *= 15 (11 female); mean age = 27.52 years, *SD* = 3.13) semester students. Hence, students in the control group and intervention group came from different semesters. However, we know from previous data collections that there are no processing or performance differences between these two semesters (cf. Castner et al. 2018), which if anything, would work against our hypotheses anyway. The data of the control group and the pre-tests of the intervention group were collected as part of a larger study where we investigated the development of visual expertise in a longitudinal study design involving all dentistry study semesters.

### Materials and apparatus

#### OPTs

**Fig. 1:**
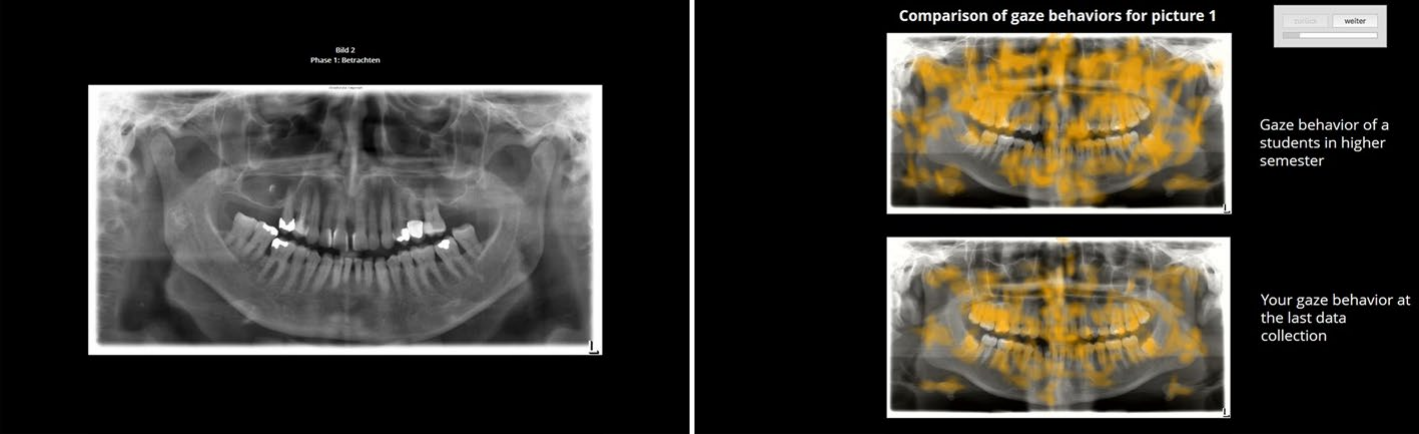
OPT displayed on a laptop (left panel) and compare-and-contrast training intervention for one OPT (right panel)

Overall, 20 OPTs that were recorded during routine checks in the university hospital were used as test and intervention stimuli. The OPTs were grouped into two sets of 10 OPTs each (set A and B). Set A was further separated into two sets A1 and A2 with five OPTs each. For the comparison between the control and intervention group, we used set A in the pre-test, set A1 and B in the post-test. Three OPTs showed no anomalies, whereas the other OPTs showed between 1 and 26 anomalies. Set A contains 79 central anomalies and 16 peripheral anomalies. Set B contains 41 central anomalies and 9 peripheral anomalies. Two experts (a maxillofacial radiologist and a prosthodontist both with over 13 years of clinical experience; co-authors of this paper) examined the OPTs and created solution templates. The OPTs had a sufficient clinical image quality (without positioning errors) and were displayed with a size between 1362 × 750 pixels and 1552 × 750 pixels (constant height for all OPTs) on a laptop (see Fig. 1, left panel).

#### Training intervention

The compare-and-contrast training intervention contained heat map combinations for 5 OPTs. For every heat map combination, two heat maps were presented among each other (see Fig. 1, right panel). The upper heat map represented the gaze behavior of an advanced student (peer-model) searching for anomalies. The lower heat map showed the current participant’s gaze behavior recorded during the pre-test. The heat map comparison had the title ‘comparison of gaze behavior for picture 1’. The peer-model was labeled as the ‘gaze behavior of an advanced student’. The participant’s heat map was labeled by ‘your gaze behavior at the last data collection’.

#### Heat maps

The heat maps were constructed using the software Eyetrace (Kübler et al. 2015). The heat maps illustrated fixations and the duration of saccades where their location and intensity was displayed in orange (see Fig. 1, right panel). Five individual heat maps showing students’ individual gaze during OPT inspection in the pre-test from set A2 were generated for each student. If students had not participated in the pre-test or had low eye-tracking quality, their recordings obtained in previous session (approx. 2 months before the pre-test) were used to create the heat maps. The five peer-model heat maps were created for the same OPTs as those used for the individual heat maps. We selected them from students who showed a full coverage and intense processing of all relevant areas of the OPTs, especially for peripheral areas.

#### Apparatus

The laptops were equipped with RED 250 mobile eye trackers (250 Hz) from SensoMotoric Instruments (SMI™). The displays (15.6 in. and resolution of 1920 × 1080 pixels) were set to the highest brightness level. In combination with a constant testing environment (room illuminance in the experimental room measured by a radiological light sensor, Gossen Mavomax™ illuminance sensor), we achieved an illumination condition of 30–40 lx on all displays. The default settings of the SMI Software BeGaze were used to classify the gaze measures (velocity-based algorithm: peak velocity 40°/s, min. fixation duration 50 ms).

### Measures

#### Diagnostic performance

The diagnostic performance was measured by evaluating students’ markings, which the students drew on the OPTs using the laptop’s mouse to control a digital pen. To assess students’ diagnostic performance in the pre- and the post-test, students first saw an OPT for 90 s. Then, they were asked to mark those regions where either treatments or further follow-up diagnostic procedures would be warranted. The markings of the students (i.e., circles drawn around suspicious regions) were saved for each OPT and were rated by two trained raters. The raters evaluated students’ markings relative to a solution template developed by two experts. The interrater reliabilities for the trained raters compared to an experienced rater were calculated for 20% of the OPTs. The agreement for each of the trained raters compared to the experienced rater for set A (for detection rate: Krippendorff’s alphas = 0.97; 0.98, for number of false positives: Krippendorff’s alphas = 0.98; 0.94) and set B (for detection rate: Krippendorff’s alphas = 0.91; 0.89, for number of false positives: Krippendorff’s alphas = 0.96; 0.95) was high, so that the two trained raters continued to code the markings independently. We used the detection rate (percentage of correctly detected anomalies) and the number of false positive markings for the analysis in the pre- and the post-test. The detection rate and the number of false positives were subdivided into two different categories—central and peripheral—depending on their location in the OPT.

#### Gaze measures

Areas of interest (AOIs) were defined for gaze behavior analysis. The anomaly-AOIs represent the anomalies in the OPTs. The anomaly-AOIs corresponded to the anomalies as they were marked in the solution template by the experts and could be further categorized as located in either peripheral or central areas. If very small anomalies located next to each other represented the same problem (e.g., cavities affecting multiple teeth), they were merged into one larger anomaly-AOI. We used the following gaze measures to analyze the eye-tracking data: the number of fixations on AOIs, fixation time in milliseconds on AOIs, time to first fixation in milliseconds on AOIs, and the overall coverage rate of the OPTs. The latter was determined by dividing each OPT into a grid that consisted of even-sized, rectangular AOIs. For smaller OPTs, we used 14 × 11 rectangular AOIs to build the grid and for bigger OPTs, we used 15 × 11 rectangular AOIs. The area of a single rectangular AOI was 6695 pixels. The coverage rate was determined as the percentage of AOIs fixated within an OPT’s grid.

#### Conceptual knowledge

The two dental medicine experts in the project developed a screening questionnaire to examine students’ baseline level of clinical knowledge in dental medicine. The majority of the items came from the Dental School’s test item repository and are used in the students’ assessments. Newly developed or modified items were reviewed by colleagues from the dental department to further ensure the items’ correctness and appropriateness. The questions were presented on the laptops with the web-based survey software tool Qualtrics. For the 20 multiple-choice questions there were four alternatives and one correct option (e.g., ‘Which answer is correct? An apical periodontitis …’ answer: ‘…points towards an endodontic problem.’). There was always one option of ‘I cannot answer the question yet/I do not know’. Students got one point for every correct answer and zero points for incorrect answers. The maximum total score was 20 points. Performance was converted into percentage correct.

### Procedure

The data collection took place in the Tübingen Digital Teaching Lab at the Leibniz-Institut für Wissensmedien between July 2017 and May 2018. In the intervention group, the pre-tests were conducted approx. 3 months before the training intervention; the post-test followed immediately after the training intervention. The delays between pre- and post-test in the control group and intervention group were the same. Data collection took place in parallel sessions with up to 30 participants, who worked individually and silently on their assignments. Ethics approval for the study was obtained from the institute’s local ethics committee (LEK 2017/016).

At the beginning of all test sessions, the students received written information on the procedure of the experiment and signed a consent form. For the diagnostic task, the students were instructed to seat themselves comfortably in front of the eye-tracker and to not move their head during the task. Then, the students were calibrated with a 13-point calibration before they received the instruction for the diagnostic task. They passed through a short drawing tutorial explaining how to mark anomalies in the OPTs using the drawing plugin tool for Mozilla Firefox™ Browser. Afterwards, the students were informed that they would see the OPTs twice, once in a search and once in a marking phase and were instructed to mark those regions that would require either treatment or follow-up diagnostic procedures. The students also saw instructions regarding cases they should not mark (missing teeth, sufficient treatments, generalized horizontal bone loss, and technical artifacts). Before students entered the search phase, they were shown a fixation cross for 2 s. In the search phase, the students were asked to look at the OPTs and search for anomalies. Each OPT was presented for 90 s. The search phase was followed by a short instruction reminding the students what anomalies to mark. In the marking phase, the students were asked to mark the detected anomalies with the drawing tool in the OPT. The procedure (instruction - fixation cross - searching phase - instruction - marking phase) was repeated for every OPT.

In the pre-test, the students performed the diagnostic task (10 OPTs of set A for the control group and 20 OPTs of set A and B for the intervention group) followed by the conceptual knowledge test.

Before the post-test, the intervention group received the compare-and-contrast modeling intervention. The students were told that they would see heat map visualizations of their gaze behavior and that of another peer student, where the intensity and location of eye movements were marked in orange. Additionally, the students in the intervention group were informed that a full coverage of OPTs is important and were instructed to compare the peer-model’s heat map in the upper part of the screen to their own individual heat map in the lower part. The verbatim instructions were as follows: ‘Please use the heat maps to compare your gaze behavior on the OPTs with the gaze behavior of a student in a higher semester. Try to identify similarities and differences in gaze behavior. […] On the next page, you can see the gaze behavior of the student in a higher semester (above) and your own gaze behavior on picture 1 (below). Please look at and compare the two heat maps. You can take as much time as you need. If you then click on ‘continue’, you will see image 1 (*the OPT*) without heat maps in full size, so that you can view it again. This process will be continued for four more images (*OPTs*). You do not have to mark any conspicuities at this point.’ Students were asked to perform the compare-and-contrast task for a total of five heat map combinations with OPTs of set A2. After the training intervention, students were asked whether they had seen differences between their own and the peer-model’s heat map. If they had seen any differences, they were asked to briefly describe these differences.

In the post-test, the students performed the diagnostic task (15 OPTs of set A1 and set B for the intervention group and 20 OPTs of set A and B for the control group) followed by the conceptual knowledge test. The students in both groups were recalibrated after five OPTs and could take a short break after 10 OPTs if they wanted.

### Data analysis

#### Missing data

One student in the intervention group had missing values in the conceptual knowledge test due to technical problems. We replaced the missing value of this student by the average group value for semester and time (pre- vs. post-test). All data points available were considered for replacement.

In addition, due to technical problems, the diagnostic performance data of single OPTs were not available for two participants of the control group. Again, we used the remaining data to estimate diagnostic performance values that were used to replace missing values.

#### Exclusion criteria

For the analysis of the gaze measures, we excluded the first fixation, which is usually residual behavior from the prior fixation cross stimuli before each OPT. Moreover, we excluded the eye tracking data of OPTs with a tracking ratio below 80%. Eye-tracking data of students who reached a tracking ratio above 80% only in half of the OPTs in the pre- or the post-test were excluded from the pre- and the post-test (*N* = 6 in control group, *N* = 6 in intervention group). Therefore, there were data from 32 participants in the intervention group and 17 participants in the control group left for analyses of gaze measures.

#### Analyses

We used linear mixed models to examine the gaze behavior (Hypotheses 1, 2a, 2b and 2c) and generalized linear mixed models for the diagnostic performance (Hypotheses 3). The R package lme4 (Bates et al. 2015) was used for the analysis. The models consisted of the same basic model structure:

$$\begin{aligned} {\text{y}}_{\text{ijkl}} & = \beta_{0} + \beta_{ 1} {\text{Time}}_{\text{ij}} + \beta_{ 2} {\text{Group}}_{\text{ik}} + \beta_{ 3} {\text{Location}}_{\text{il}} + \beta_{ 4} \left( {{\text{Time}} \times {\text{Group}}} \right)_{\text{ijk}} \\ & + \,\beta_{ 5} \left( {{\text{Time}} \times {\text{Location}}} \right)_{\text{ijl}} + \beta_{ 6} \left( {{\text{Group}} \times {\text{Location}}} \right)_{\text{ikl}} + \beta_{ 7} \left( {{\text{Time}} \times {\text{Group}} \times {\text{Location}}} \right)_{\text{ijkl}} \\ & + \,\beta_{ 8} {\text{Conceptual Knowledge}}_{\text{i}} + v_{{0{\text{i}}}} + v_{{ 1 {\text{i}}}} {\text{Time}}_{\text{ij}} + \varepsilon_{\text{ijkl}} \\ \end{aligned}$$

y_ijkl_ represents the gaze measure/diagnostic performance of student i. *β*_0_ specifies the intercept across students for the reference categories. The effect of time *β*_1_ (pre-/post-test), the effect of group *β*_2_ (control/intervention), and the effect of location *β*_3_ (central/peripheral anomalies) were included to test the main effects. *β*_4_, *β*_5_, and *β*_6_ each represent two-way interactions between time, group and location; *β*_7_ specifies the three-way interaction between time, group, and location. The effect of the intervention on peripheral and central anomalies was tested by the three-way interaction. With *β*_8_, conceptual knowledge is included as a covariate; *v*_0i_ specifies the individual intercept for each student and *v*_1i_ the individual slope over time for each student, see also “Appendix 1” for the measures. Adjustments were made in cases where additional factors had to be included. We used *d* as an effect size, with *d* = .20 to .40, *d *= .50 to .70, and *d* > .80 corresponding to small, medium and large effects, respectively (Cohen 1988).

#### Data transformation

Data distributions of gaze measures were checked by graphical methods (quantile—quantile plots and scatter plots for residuals and predicted values). We used log-transformed values for fixation time and number of fixation (Hypotheses 2a and 2b) because the scatter plots for residuals and predicted values showed a better distribution for log-transformed values than original values (see “Appendix 2”). For all other measures and analyses, we used the original values due to better distribution in the scatter plot.

## Results

### Comparison between the control and intervention group

#### Visual coverage of the OPTs (Hypothesis 1)

To test whether the compare-and-contrast intervention would affect the coverage of the OPTs, we augmented the aforementioned basic model with the random factor OPT to account for differences between the OPTs. Moreover, we excluded the factor specifying the location of anomalies and its interactions from the analysis, since the analyses referred to the OPT as a whole rather than to the anomalies contained within them (see “Appendix 3”).

In line with our hypothesis, the results indicated a significant interaction between time and group, Estimate = 4.08, *t*(52) = 2.50, *p* = .02 (*d* = .36). The coverage rate for the intervention group increased slightly from pre- to post-test; however, the coverage rate decreased for the control group (see Table 1). Moreover, students’ conceptual knowledge affected their coverage in that better conceptual knowledge was related to a higher coverage rate, Estimate = .45, *t*(87) = 2.53, *p* = .01 (*d* = .36).

**Table 1:** Means and standard deviation of gaze coverage rates

	Control group		Intervention group	
	Pre-test	Post-test	Pre-test	Post-test
*Gaze coverage rate (%)*				
Mean	49.03	47.09	47.62	48.61
SD	6.65	6.38	6.31	7.42

#### Gaze behavior regarding anomalies (Hypotheses 2a, 2b, 2c)

**Fig. 2:**
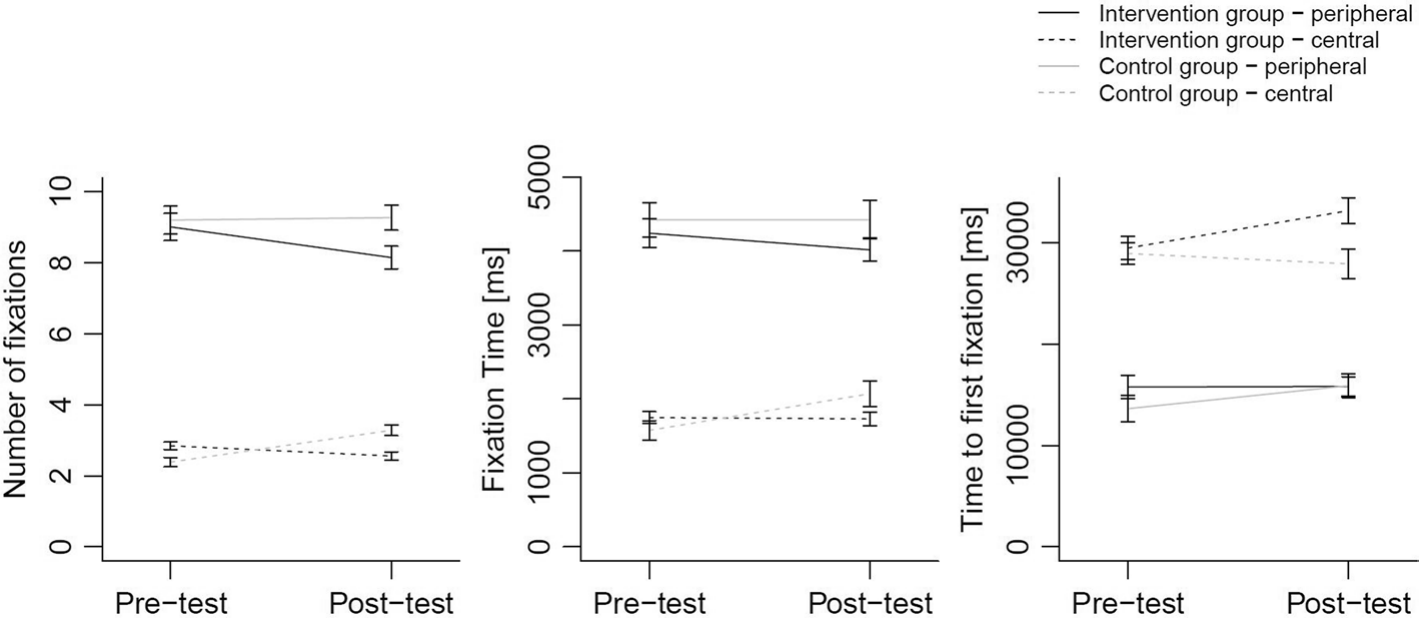
Means and standard errors of number of fixations (left panel), fixation time (middle panel) and time to first fixation of anomalies (right panel) for groups (intervention vs. control), time (pre-test vs. post-test) and location (central vs. peripheral anomalies)

*Number of fixations (Hypothesis 2a)* The analysis for number of fixations revealed a three-way interaction between time, group and location, Estimate = .33, *t*(94) = 3.01, *p* = .003 (*d *= .43), that was, however, not in the expected direction (see “Appendix 3”). Contrary to our assumption, the number of fixations did not increase for peripheral anomalies in the intervention group. Rather, for both central and peripheral anomalies, the number of fixations decreased in the intervention group, whereas it increased in the control group (see Fig. 2, Table 2). This effect was stronger for peripheral anomalies than for central anomalies.

**Table 2:** Means and standard deviations of gaze measures and diagnostic performance

	Control group				Intervention group			
	Pre-test		Post-test		Pre-test		Post-test	
	Central	Peripheral	Central	Peripheral	Central	Peripheral	Central	Peripheral
*Number of fixations*								
Mean	2.39	9.21	3.28	9.28	2.85	9.01	2.56	8.15
SD	0.54	1.62	0.61	1.45	0.63	2.14	0.65	1.80
*Fixation time (ms)*								
Mean	1575.60	4424.18	2070.65	4423.40	1747.96	4241.70	1731.52	4016.95
SD	539.68	956.70	713.06	1075.59	456.05	1098.39	518.40	884.93
*Time to first fixation (ms)*								
Mean	28,927.50	13,627.18	27,910.00	15,889.39	29,471.55	15,764.38	33,156.58	15,813.96
SD	4462.30	5372.14	5967.49	4836.07	6506.46	6501.21	7250.19	5356.15
*Detection rate (%)*								
Mean	51.51	47.06	50.59	51.93	51.69	54.02	54.70	57.02
SD	8.95	11.63	12.52	14.85	9.85	14.85	10.73	18.79
*Number of false positive markings per OPT*								
Mean	1.12	0.45	1.12	0.55	1.01	0.36	1.54	0.74
SD	0.58	0.47	0.71	0.45	0.56	0.26	0.89	0.60

*Fixation time (Hypothesis 2b)* We did not find the expected three-way interaction for increase in fixation time on peripheral anomalies due to the intervention. Nevertheless, separate effects for location and an interaction between time and group were found: Fig. 2 and Table 2 show that students fixated on peripheral anomalies longer than central anomalies, Estimate = 1.09, *t*(94) = 13.02, *p* < .001 (*d* = 1.95). Contrary to our hypothesis, the interaction effect between time and group, Estimate = −.31, *t*(113) = −2.66, *p* = .009 (*d *= .37), was in the opposite direction. In the intervention group the fixation time on anomalies slightly decreased, whereas the fixation time on anomalies increased in the control group.

*Time to first fixation (Hypothesis 2c)* For the time to first fixating on an anomaly, results revealed the expected significant three-way interaction between time, group and location, Estimate = −6915.16, *t*(141) = −2.14, *p* = .03 (*d *= .31). Figure 2 and Table 2 show that students in the intervention group fixated on central anomalies in the post-test later than in the pre-test. In contrast, students in the control group fixated on central anomalies in the post-test sooner compared to the pre-test. This pattern tended to reverse for peripheral anomalies in that students fixated on them earlier after the intervention.

#### Diagnostic performance (Hypothesis 3)

We used a binomial distribution to analyze detection rate and a Poisson distribution to analyze the number of false positive markings. The model was the same as indicated in “Appendix 1”.

**Fig. 3:**
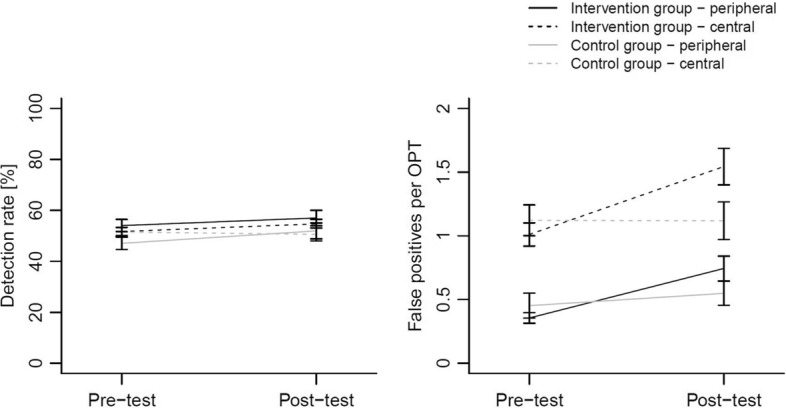
Means and standard errors of detection rate (left panel) and false positive markings per OPT (right panel) for groups (intervention vs. control), time (pre-test vs. post-test) and location (central vs. peripheral anomalies)

*Detection rate* Against our hypothesis, results did not show an improvement of the detection rate for peripheral anomalies in the intervention group. However, the results revealed a significant interaction between time and group, Odds ratio (OR) = .26, *z *= 2.12, *p* = .03 (*d *= .28). The chance to detect anomalies independent of location slightly increased due to the intervention, but remained stable in the control group (see “Appendix 4”). Figure 3 and Table 2 show that this significant interaction was triggered by a slight decrease in the control group for central anomalies.

*Number of false positive markings* In general, students made more false positive markings in the central area than in the periphery, Estimate = −0.91, *z* = −7.88, *p* < .001 (*d* = .97) (see “Appendix 4”). Contrary to our assumptions, the interaction between time and group showed that the chance to make false positive markings increased in both the intervention and control group, Estimate = .47, *z* = 2.95, *p* = .003 (*d *= .38). However, the increase was stronger in the intervention group (see Fig. 3, Table 2).

### Exploratory analyses

As our intervention did not lead to meaningful improvements of diagnostic performance, we further explored the data to shed light on potential reasons for this finding. One reason could be that our gaze-based intervention only addressed detection errors while recognition and decision-making errors may have occurred as well. To investigate this presumption, we analyzed the distribution of errors resulting from bottom-up processes (detection errors) and errors resulting from top-down processes (recognition and decision-making errors) of all students in the post-test.

Detection errors referred to cases where students neither fixated on nor marked an anomaly. Recognition and decision-making errors were qualified by at least one fixation on an anomaly in combination with a missing marking of that anomaly. The analysis of error types showed that students made on average 0.58 (16%) detection errors and 3.12 (84%) recognition and decision-making errors per OPT. Therefore, students made over five times more errors resulting from top-down than bottom-up processes.

## Discussion

The aim of the study was to improve dental students’ search behavior and diagnostic performance in reading OPTs by means of a gaze-based compare-and-contrast modeling intervention. Based on previous research and theories, we hypothesized that students often commit detection errors during OPT interpretation, which could be avoided by supporting them in fuller visual coverage of an OPT. Therefore, students were asked to compare and contrast heat maps of gaze visualizations of a model showing a full gaze coverage and their own heat maps. With this intervention, we aimed at improving students’ gaze coverage of OPTs, thereby reducing detection errors.

According to Hypothesis 1, we expected that the gaze coverage of OPTs would increase due to the intervention. Our results support this hypothesis; however, the effect was rather small with a slight increase in the intervention group and a decrease in the control group. In fact, the groups differed regarding coverage of OPTs contained in the post-test only by 1.5%. A reason for this finding may be that our intervention used a very implicit way of increasing gaze coverage. Previous research showed that rather explicit full coverage trainings lead to about 7% difference between control and training group (Kok et al. 2016). Nevertheless, a small increase like the one found in the current study for a short gaze-based intervention with only five heat map comparisons could be also meaningful and a first step to increase coverage when applied over a longer duration or combined with more explicit instruction as to how to compare the heat maps.

For the search behavior (Hypotheses 2a, 2b, 2c), we assumed that students would fixate on peripheral anomalies more often, for longer, and sooner after the intervention. Most of these predictions were not confirmed; nevertheless, students changed their gaze behavior after they had received the intervention. They fixated on anomalies independent of location less often and for shorter periods of time. A reason for the unexpected behavior could be that students tried to cover OPTs fully, in that they expanded their visual attention, which could be associated with fewer and shorter fixations. The literature on expertise development is ambiguous when it comes to fixation times and number of fixations. Van der Gijp et al. (2017) found that studies equally often report either a decrease or increase of fixation time and number of fixations on relevant areas with higher expertise level. Thus, advanced gaze behavior may be reflected in very different eye tracking result patterns. On the one hand, the fact that students visually process the anomalies less intensely could mean that they were overly occupied with inspecting other areas of the OPTs to obtain full coverage. Therefore, students may not have had enough time to process anomalies sufficiently, leading to a negative diagnostic outcome. On the other hand, it is yet unclear how intensive the visual processing of anomalies must be in order to gain a good diagnostic outcome. Shorter and fewer fixations could mean that students only decided faster. Thus, the intervention lead to a change in the number of fixations and fixation time on anomalies, but further research is needed to specify whether these changes reflect either more efficient or inadequate processing.

The results of the current study confirmed Hypothesis 2c. The intervention lead to later fixations on central anomalies and sooner fixations on peripheral anomalies. Although the effect was small, we can see that the intervention shifts attention towards the peripheral areas of OPTs.

We expected that the intervention would improve diagnostic performance especially, in peripheral areas, as more attention would be directed at these areas (Hypothesis 3). We found that students detected more anomalies independent of location due to the intervention. However, this increase was small, and we are reluctant to interpret this effect as a meaningful improvement. Additionally, an increase in the detection of central anomalies in the control group seems to drive the effect. Thus, we conclude that the intervention did not lead to a meaningful improvement of anomaly detection. Potential reasons for this pattern of results could be traced back to either (a) the only small effects of the intervention on gaze coverage or (b) the type of errors students make:

With the compare-and-contrast intervention, we addressed detection errors, which are errors caused by overlooking anomalies. Our results showed that the intervention lead to only a small increase in OPT coverage, which could be a reason for the very small improvements in detection rate. Another reason might be that—different from what we had expected based on the literature (e.g., Donald and Barnard 2012)—students do not struggle the most with detection errors, but with recognition and decision-making errors, which were not addressed with our intervention. To investigate this post hoc explanation, we explored the frequency of detection errors (bottom-up processes) and recognition and decision-making errors (top-down processes) students made during OPT inspection. The results showed that students made about five times more recognition and decision-making errors than detection errors. This large difference could explain why the detection of anomalies did not improve substantially, since our intervention had addressed only a small part of all errors that students made. Thus, future studies in the field of dental radiology should focus more strongly on how to prevent top-down errors (recognition and decision-making), which may be caused by a lack of knowledge about the pathology and the visual characteristics of anomalies. In line with this assumption, research showed that students who learned basic biomedical knowledge improved diagnostic performance of dental radiographs (Baghdady et al. 2009). These observations also reflect the contentious points of theoretical considerations. For decades, two different approaches of problem-based learning in medical education—teaching a problem solving process versus teaching knowledge—have been discussed (cf. Servant-Miklos 2019). It is still an open question whether teaching a problem solving process—as we did in this intervention—or teaching knowledge is more beneficial for students (Schmidt and Mamede 2015). First evaluations suggest that teaching knowledge is more effective (Monteiro et al. 2020; Schmidt and Mamede 2015). These results also support the view that further studies should focus on improving knowledge in the interpretation of radiographs.

Contrary to our expectations, we found no improvement (decrease) for the marking of false positive errors but an increase caused by the intervention. Students in the intervention group even marked more false positives, whereas students in the control group did not change in committing false positive errors from pre- to post-test. A possible explanation could be that students in the intervention group felt encouraged to find more anomalies. However, due to their potential lack of knowledge regarding characteristic features of anomalies, they did not find more true positive anomalies. Instead, they defined other areas as conspicuous, which resulted in more false positive markings. This phenomenon that interventions lead to more false positive errors is also known in literature (Ganesan et al. 2018). Swensson et al. (1977, 1985) found that searching for specific anomalies or searching in specific areas lead to an increase in false positive rates. Ganesan et al. (2018) explain this phenomenon by assuming that an intervention may interrupt regular search behavior and therefore lead to more errors.

### Limitations

The study has some limitations regarding methods and design. First, five OPTs were used in the pre- and the post-test, which could affect the performance in line with the testing effect (Roedinger and Karpicke 2006). However, a potential testing effect should affect the control group in similar ways, but we did not find any improvements for diagnostic performance there. Additionally, previous studies found that observers do not remember the radiographs correctly, suggesting that their repeated use may have little, if any effect on diagnostic performance (Hillard et al. 1985; Myles-Worsley et al. 1988; Ryan et al. 2011).

Second, the rather small sample size in the current study could have contributed to the small effects. However, compared to previous expertise studies that investigated visual search in medical image processing with, on average, only six to eight participants (cf. Gegenfurtner et al. 2011), the sample size in the current study (*N* = 61) is substantial in terms of its statistical power.

Third, the quasi-experimental design with non-randomized groups presents a major limitation of the present study. Due to this design, we cannot exclude that the effects were influenced by cohort differences. However, data from our longitudinal study indicate that the diagnostic performance and most of the gaze measures do not differ between the cohorts. Unfortunately, a randomization of students was not feasible due to different and full schedules of the students. Moreover, the fact that they had to attend the study twice complicated the management and may have decreased students’ motivation to participate in the study anyway.

Fourth, the intervention was designed to provide a rather general level of gaze guidance, which may not have been sufficiently specific to improve students’ performance. In contrast, dynamic gaze guidance, where attention is directed towards relevant areas on a moment-to-moment basis, has been shown to foster the diagnostic performance of observers (Litchfield et al. 2010; Gegenfurtner et al. 2017). Moreover, dynamic gaze guidance offers information regarding the sequence of inspecting regions and illustrates detailed search strategies. The absence of this information could also have contributed to the pattern of results in the present study. Therefore, it would be worth to investigate in future research, whether dynamic gaze guidance can be helpful for dental students when learning to read OPTs.

### Conclusion and implications

In this study, we investigated the effects of a gaze-based intervention on gaze behavior and diagnostic performance in dental students reading OPTs. The intervention changed the gaze behavior of dental students by changing their visual attention but did not improve their diagnostic performance. A potential reason for these findings is that the intervention was developed to address students’ detection errors, while post hoc exploratory analyses showed that students committed more recognition and decision-making errors than detection errors. Thus, interventions focusing only on a full coverage of radiographs appear to not offer the appropriate level of support students would need to improve their diagnostic performance. An alternative training approach would be to focus on teaching visual characteristics of anomalies and basic knowledge of relevant pathology, thereby facilitating top-down processes that help to avoid recognition and decision-making errors (cf. Kok and Jarodzka 2017b).

## Appendix 1

See Table 3.

**Table 3:** Parameters and their explanation of the model for Hypotheses 1, 2a, 2b, 2c and 3

Parameters	Explanation of parameters
y_ijkl_	Estimated value for each student i, at time j in the specific group k, for the location l while considering the conceptual knowledge of student i
*β*_0_	Intercept across students for the reference categories (pre-test, control group, central anomalies)
*β*_1_	Fixed effect of time (pre- vs. post-test)
*β*_2_	Fixed effect of group (control vs. intervention group)
*β*_3_	Fixed effect of location (central vs. peripheral anomalies)
*β*_4_	Interaction of time and group
*β*_5_	Interaction of time and location
*β*_6_	Interaction of group and location
*β*_7_	Three-way interaction of time, group and location
*β*_8_	Conceptual knowledge as covariate to control for different levels
*v*_0i_	Random effect: individual intercept for each student
*v*_1i_	Random effect: individual slope over time for each student
ε_ijkl_	Error term

## Appendix 2

See Table 4.

**Table 4:** Means and standard deviations of gaze measures for log-transformed values

	Control group				Intervention group			
	Pre-test		Post-test		Pre-test		Post-test	
	Central	Peripheral	Central	Peripheral	Central	Peripheral	Central	Peripheral
*Number of fixations*								
Mean	0.84	2.21	1.17	2.22	1.02	2.17	0.91	2.07
SD	0.26	0.18	0.19	0.16	0.22	0.25	0.25	0.23
*Fixation time (ms)*								
Mean	7.28	8.37	7.58	8.37	7.43	8.31	7.42	8.28
SD	0.48	0.22	0.35	0.24	0.27	0.29	0.29	0.22

## Appendix 3

See Table 5.

**Table 5:** Model parameters from the linear mixed models of coverage and gaze measures

	Gaze coverage rate		Number of fixations		Fixation time		Time to first fixation	
	Estimate (SE)	t value (*df*)	Estimate(SE)	t value (*df*)	Estimate (SE)	t value (*df*)	Estimate (SE)	t value (df)
*Fixed effects*								
Intercept	43.54 (2.25)	t(83.81) = 19.35***	0.89 (0.09)	t(109.11) = 9.42***	7.26 (0.12)	t(105.85) = 58.56***	25,987.05 (2459.31)	t(122.99) = 10.57***
Time^a^	−2.85 (1.41)	t(61.47) = −2.02*	0.34 (0.07)	t(127.78) = 4.88***	0.29 (0.10)	t(118.13) = 3.04**	−1893.38 (1943.07)	t(154.67) = −0.97
Group^b^	−0.85 (1.31)	t(47.81) = −.65	0.17 (0.07)	t(91.12) = 2.47*	0.15 (0.09)	t(86.91) = 1.63	896.21 (1864.53)	t(106.35) = 0.48
Location^c^			1.36 (0.06)	t(94.00) = 21.40***	1.09 (0.08)	t(94.00) = 13.02***	−15,300.32 (1846.38)	t(140.82) = −8.29***
Conceptual knowledge	0.45 (0.18)	t(86.71) = 2.53*	0.00 (0.01)	t(86.50) = −.062	0.00 (0.01)	t(80.02) = 0.22	265.89 (176.59)	t(106.08) = 1.51
Time × group	4.08 (1.63)	t(51.92) = 2.50*	−0.45 (0.08)	t(122.69) = −5.36***	−0.31 (0.12)	t(112.66) = −2.66**	5441.30 (2346.03)	t(146.71) = 2.32*
Time × location			−0.32 (0.09)	t(94.00) = −3.54***	−0.30 (0.12)	t(94.00) = −2.57*	3279.71 (2611.17)	t(140.82) = 1.26
Group × location			−0.22 (0.08)	t(94.00) = −2.73**	−0.21 (0.10)	t(94.00) = −2.02*	1593.15 (2284.78)	t(140.82) = 0.70
Time × group × location			0.33 (0.11)	t(94.00) = 3.01**	0.28 (0.15)	t(94.00) = 1.91	−6915.16 (3231.16)	t(140.82) = −2.14*
*Random effects*								
Individual intercept (SD)	4.00		0.14		0.19		3001.87	
Individual slope over time (SD)	4.77		0.08		0.16		690.32	
Correlation of intercept and slope	−0.35		−0.67		−0.77		−1.00	
Residual variance (SD)	4.83		0.19		0.24		5383.07	
Individual intercept for OPTs (SD)	1.40							

^a^Dummy-coded (pre-test as reference category)

^b^Dummy-coded (control group as reference category)

^c^Dummy-coded (central anomalies as reference category)

**p* < .05, ***p* < .01, ****p* < .001

## Appendix 4

See Table 6.

**Table 6:** Model parameters from the generalized linear mixed models of diagnostic performance

	Detection rate		Number of false positive markings	
	Estimate (SE)	z value	Estimate (SE)	z value
*Fixed effects*				
Intercept	− 0.25 (0.18)	− 1.37	2.42 (0.22)	10.78***
Time^a^	− 0.14 (0.10)	− 1.42	0.32 (0.13)	2.42*
Group^b^	0.05 (0.09)	0.55	− 0.13 (0.13)	− 0.99
Location^c^	− 0.18 (0.11)	− 1.60	− 0.91 (0.12)	− 7.88***
Conceptual knowledge	0.03 (0.01)	1.85	− 0.01 (0.02)	− 0.29
Time × group	0.26 (0.12)	2.12*	0.47 (0.16)	2.95**
Time × location	0.23 (0.16)	1.47	0.20 (0.15)	1.39
Group × location	0.28 (0.14)	1.93	− 0.14 (0.15)	− 0.90
Time × group × location	− 0.23 (0.20)	− 1.15	0.11 (0.19)	0.61
*Random effects*				
Individual intercept (SD)	0.25		0.38	
Individual slope over time (SD)	0.31		0.43	
Correlation of intercept and slope	0.10		− 0.21	

^a^Dummy-coded (pre-test as reference category)

^b^Dummy-coded (control group as reference category)

^c^Dummy-coded (central anomalies as reference category)

**p* < .05, ***p* < .01, ****p* < .00
