# Correction to: How to support dental students in reading radiographs: effects of a gaze‑based compare‑and‑contrast intervention

**DOI:** 10.1007/s10459-021-10058-7

**Published:** 2021-06-28

**Authors:** Thérése F. Eder, Juliane Richter, Katharina Scheiter, Constanze Keutel, Nora Castner, Enkelejda Kasneci, Fabian Huettig

**Affiliations:** 1 Leibniz-Institut für Wissensmedien, Schleichstraße 6, 72076 Tübingen, Germany; 2 University of Tübingen, Tübingen, Germany; 3 Department of Oral‑ and Maxillofacial Radiology, Centre for Dentistry, Oral Medicine, and Maxillofacial Surgery, University Hospital Tübingen, University of Tübingen, Tübingen, Germany; 4 Perception Engineering, Department of Computer Science, University of Tübingen, Tübingen, Germany; 5 Department of Prosthodontics, Centre for Dentistry, Oral Medicine, and Maxillofacial Surgery, University Hospital Tübingen, University of Tübingen, Tübingen, Germany

## Correction to: Advances in Health Sciences Education (2021) 26:159–181 https://doi.org/10.1007/s10459-020-09975-w

The article “How to support dental students in reading radiographs: effects of a gaze‑based compare‑and‑contrast intervention”, written by “Thérése F. Eder, Juliane Richter, Katharina Scheiter, Constanze Keutel, Nora Castner, Enkelejda Kasneci, Fabian Huettig”, was originally published Online First without Open Access. After publication in volume 26, issue 1, page 159–181 the author decided to opt for Open Choice and to make the article an Open Access publication. Therefore, the copyright of the article has been changed to © The Author(s) 2021 and the article is forthwith distributed under the terms of the Creative Commons Attribution 4.0 International License, which permits use, sharing, adaptation, distribution and reproduction in any medium or format, as long as you give appropriate credit to the original author(s) and the source, provide a link to the Creative Commons licence, and indicate if changes were made.

The images or other third party material in this article are included in the article’s Creative Commons licence, unless indicated otherwise in a credit line to the material. If material is not included in the article’s Creative Commons licence and your intended use is not permitted by statutory regulation or exceeds the permitted use, you will need to obtain permission directly from the copyright holder.

To view a copy of this licence, visit http://creativecommons.org/licenses/by/4.0/

